# Novel Nanocrystal Injection of Insoluble Drug Anlotinib and Its Antitumor Effects on Hepatocellular Carcinoma

**DOI:** 10.3389/fonc.2021.777356

**Published:** 2021-12-02

**Authors:** Mei Luo, Huiwei Sun, Qiyu Jiang, Yantao Chai, Congshu Li, Bin Yang, Zhixian Hong

**Affiliations:** ^1^ Institute of Life Sciences, Jinzhou Medical University, Jinzhou, China; ^2^ Department of Infectious Disease, The Fifth Medical Center of Chinese PLA General Hospital, Beijing, China; ^3^ Department of Hepatology, The Fifth Medical Center of Chinese PLA General Hospital, Beijing, China; ^4^ Department of Hepatobiliary Surgery, The Fifth Medical Center of Chinese PLA General Hospital, Beijing, China

**Keywords:** advanced hepatocellular carcinoma, molecularly targeted agents, anlotinib, nanoscale crystal injection, antitumor activation

## Abstract

The molecularly targeted agent anlotinib offers a novel therapeutic strategy against advanced hepatocellular carcinoma (HCC). With this study, we aimed to solve the technical problem of anlotinib being insoluble in injectable solutions; we also aimed to assess the antitumor activity of anlotinib on hepatocellular carcinoma cells. We prepared an anlotinib nanocrystal injection by wet grinding, and we optimized the prescription process using a transmission electron microscope (TEM) and a laser particle size analyzer (LPSA). The release of anlotinib from the injected nanocrystals was evaluated using LC-MS/MS *in vitro*, and the drug’s anti-tumor effects were assessed in a nude mice tumor model. The anlotinib nanocrystals had a uniform particle size distribution (the average nanoparticle size was ~200 nm). The preparation of anlotinib into nanocrystals did not change the original crystal structure. The intravenous injection of anlotinib nanocrystals achieved anti-tumor activity at very low doses compared to those required for oral administration of an anlotinib suspension: anlotinib nanocrystals at a dose of 50 μg/kg inhibited the subcutaneous growth of the HCC cell line MHCC97-H; whereas the dose of anlotinib suspension required for an equivalent effect was 1 mg/kg. Therefore, our novel anlotinib nanocrystal injection preparation provides an option for achieving a safe and effective molecularly targeted therapy against advanced HCC.

## 1 Introduction

Advanced hepatocellular carcinoma (HCC) is a serious physical and mental health threat in China (1–4). One of the main therapeutic strategies for advanced HCC is molecularly targeted therapy, that is, oral administration of molecularly targeted agents (5–8). These agents are small molecular tyrosine kinase inhibitors (TKIs) that directly prevent the proliferation, metastasis or, angiogenesis of tumors by inhibiting the activation of some receptor tyrosine protein kinases (including vascular endothelial growth factor receptors [VEGFRs] or platelet-derived growth factor receptors [PDGFRs]) or kinases belonging to the MAPK (mitogen-activated protein kinase) pathway (for example, B-Raf) (9–11). The first of these drugs approved for marketing was sorafenib (12–14). Since their approval, regorafenib, lenvatinib, and cabozantinib have been used for advanced HCC treatment (15–18). The results of clinical trials seem to show that the new molecularly targeted drugs such as regorafenib, lenvatinib, and cabozantinib are superior to sorafenib, but the structures of these four compounds have similar chemical structure patterns, all these drugs are modifications of a basic 1-(4-(pyridin-4-yloxy)phenyl) urea structure (15–20). Therefore, new drug candidates for treatment are needed.

Anlotinib is a newly developed and orally administrated multi-targeted TKI (21–24). Increasing evidence indicates that anlotinib may inhibit the proliferation of some human malignancies by blocking VEGFR2 phosphorylation and by its antagonist-activity against c-MET, c-Kit, Ret, Aurora-B, c-FMS, and the discoidin domain receptor 1 (DDR1) (25–27). Moreover, anlotinib was approved by the CFDA for the clinical treatment of NSCLC in 2018 (28). Therefore, anlotinib may be prove to be an effective treatment for patients with advanced HCC.

Anlotinib is usually administered to patients *via* the oral route (oral anlotinib hydrochloride capsule) (29). However, long treatment cycles (due to the limitations of anlotinib’s own chemical properties) usually lead to multi-drug resistance (MDR) of the cancer cells (30–32). Special equipment for nanocrystal preparation can be used to disperse the insoluble drug particles that are coarsely scattered in water into sub-nano dispersions of drug particles smaller than 500 nm without changing the original crystal structure of the drug-delivering system (33–36). Therefore, novel nano-preparation technologies may improve the physical and chemical properties of anlotinib. The development of anlotinib nanocrystal injections for intravenous administration should greatly improve the bioavailability of the drug to improve its anti-tumor effects with a smaller dose than the one currently required for treatment. For this study, we prepared anlotinib nanocrystals for intravenous injection; we systematically investigated the preparation process variables and prescription compositions and characterized and evaluated the obtained anlotinib nanocrystal preparations.

## 2 Materials and Methods

### 2.1 Chemical Agents Used in This Work

The pure anlotinib powder (a reference substance with an HPLC purity >98.7%) was chemically synthesized by Shuang Cao in the Wuhan Institute of Technology (Wuhan City, Hubei, China). Poloxamer 188 was purchased from the BASF Corporation (Ludwigshafen, Germany). Tween 80 was purchased from Serva (Heidelberg, Germany). The egg yolk lecithin for injections was purchased from Lipoid (Ludwigshafen, Germany). The cell line used in the study, MHCC97-H, was a gift from Dr. Yan Chen at the Center of Therapeutic Research for Liver Cancer (Beijing the 302 Hospital, Beijing, China) and has been described in previous publications (37, 38). The BalB/c nude mice used in the experiments (BalB/c strain lacking a thymus [absence of mature T cells] immunodeficiency) were purchased from Beijing-Si-bei-fu Company (Beijing China). The serum and culture medium for cell culture experiments were purchased from Thermo (USA; Invitrogen brand), and the laboratory consumables such as cell culture flasks and cell culture dishes were purchased from Corning (NY, USA). The high-performance liquid chromatograph (HPLC), transmission electron microscope (transmission electron microscope, TEM) and other equipment are products of Hitachi (Japan); the liquid chromatography-mass spectrometer (ESI/TOF/API4000) was purchased from Applied Biosystems/MDS Analytical Technologies USA Products; the laser Zeta potential particle size analyzer (Zetasizer) is a product of Malvern (United Kingdom); the wet mill is a product of the Swiss WAB-Group (Basel Region, Switzerland); and, the T25 type dispersing and emulsifying homogenizer is a product of the German IKA company (Janke & Kunkel OHG, Germany).

### 2.2 Preparation of Anlotinib Nanocrystals

We prepared the anlotinib nanocrystal injection using a ball milling method (39, 40), the specific steps were the following: We used prescribed amounts of charge and space stabilizer (poloxamer-188 and egg yolk lecithin); we added part of the prescription amount of distilled water and magnetically stirred the mixture to make it evenly dispersed; next, we added the prescribed amount of anlotinib. The concentration of anlotinib in the prepared anlotinib nanocrystal was approximately 10 mg/mL; we used the T25 high shear emulsification homogenizer at 12000 rpm for 15 minutes to disperse the crystals. Next, we poured the dispersed suspension into the grinding chamber and added the remaining water, using 0.3 mm zirconia beads as the grinding spheres. The speed of the grinding chamber was gradually increased from 1500 to 4000 rpm taking samples at 30, 60, 90, and 120 minutes and measuring the average particle sizes at each time point. When the mean particle size of anlotinib was less than 300 nm, we stopped the grinding and collected the formulation.

We prepared a solution of anlotinib to use as a control. The anlotinib solution was prepared following published methods (41–44). Briefly, the anlotinib powder was dissolved in organic solvents, PEG400, TWEEN80, and DMSO. Next, the anlotinib organic solvents solution was dissolved in PBS to a final anlotinib solution concentration of ~2 mg/ml (almost 2mg/ml).

### 2.3 Physical Characterization of Anlotinib Nanocrystals

#### 2.3.1 Anlotinib-Nanocrystal Size Distribution and Zeta Potential-Dynamic Light Scattering (DLS)

We diluted 100 μL of the above anlotinib nanocrystal suspension (see section 2.2) in distilled water and measured the particle size distribution, average particle size, or Zeta potential using a Nano-ZS90 nanometer.

#### 2.3.2 Transmission Electron Microscope Observation Using Phosphotungstic Acid Negative Staining Method

We used a transmission electron microscope to observe the microscopic morphology of the anlotinib nanocrystals. Briefly, we diluted 100 μL of the anlotinib nanocrystal suspension (see section 2.2) in distilled water and added drops to the copper grid with a supporting film. When the moisture had completely dried, we added an appropriate amount of phosphotungstic acid saturated solution. After the moisture had evaporated, we placed the sample in the machine and took pictures.

#### 2.3.3 X-Ray Powder Diffraction Analysis

The anlotinib nanocrystal was freeze-dried to remove all the water contents before testing the sample by X-ray powder diffraction. The cathode was a Cu•Kα1; the voltage was set at 220 V; the starting angle and the ending angle were set at 2.0 (θ) and 40 (θ), respectively; the emission angle and the receiving angle were 0.5° and 0.3°, respectively; the step size was 0.015; the scanning speed was set at 4°/min, and the wavelength at 1.5406/cm. After preparing the sample, we placed it in the X-ray optical path of the diffractometer and rotated it along the fixed axis; the θ angle was changed, and the diffraction pattern and data were recorded.

### 2.4 *In Vitro* Release of Anlotinib From the Nanocrystals

We poured 0.5 mL of the above anlotinib nanocrystal (10 mg/mL) suspension into a dialysis bag (45). We placed the dialysis bag in 200 mL of release medium (0.5% sodium lauryl sulfate, 0.9% sodium chloride, 20 nM phosphate buffer, pH 7.0) and kept it at 37°C on a shaker platform rotating at 50 rpm. We took 1 mL samples of release medium at different time points and replaced the volume with blank medium. Each medium sample taken was passed through a 0.22-μm microporous membrane, and the anlotinib content was determined by HPLC. The HPLC liquid phase conditions included: an Agilent ZOBAX C18 column (250 × 4.6 mm, 5 µm), the mobile phase was an acetonitrile-ammonium acetate buffer (40:60, pH 8.50 ± 0.05 adjusted with ammonia), and the flow rate was 1.0 mL•min^-1^. The detection wavelength was 247 nm, the column temperature was set at 40°C, and the injection volume was 20 μL.

For the disposition of Anlotinib-nanocrystal injection, twelve healthy male Vistar rats were taken, weighed, and randomly divided into two groups, each with 6 rats. The Anlotinib-nanocrystal injection was administered intravenously at a dose of 7 mg/kg. One group was decapitated 3h and 9h after administration for another group, blood was drained quickly, heart, liver, spleen, lung, kidney and brain tissues were taken, washed with normal saline, blotted dry with filter paper, and stored at -80°C for later use. For sample determination, take 0.5g of homogenized sample, centrifuge at 16112×g for 3min, taken 200μL of the supernatant for HPLC.

### 2.5 The *In Vivo* Tumor Experiments

We cultured MHCC97-H cells and injected them into subcutaneous tissue of nude mice to form tumors (46–49). The amount of cells injected in each animal is exactly the same (5×10^6^ cells for every mice). Next, we grouped the mice randomly. Mice in each model group received a different treatment: oral administration of crude anlotinib suspension (concentrations: 3 mg/kg, 2 mg/kg, 1 mg/kg, 0.5 mg/kg, or 0.2 mg/kg); a tail-vein injection of anlotinib nanocrystals (concentrations: 0.5 mg/kg, 0.2 mg/kg, 0.1 mg/kg, 0.05 mg/kg, or 0.02 mg/kg); and a tail-vein injection of anlotinib solution (concentrations: 0.5 mg/kg, 0.2 mg/kg, 0.1 mg/kg, 0.05 mg/kg, or 0.02 mg/kg). After 21 days of treatment (approximately 10 doses of treatment), we collected the subcutaneous tumor tissues and measured their volumes and weights according to published methods (50, 51). Each group consists of 6-10 animals, each animal has one subcutaneous tumor tissue. As a control, another three groups of nude mice were respectively inoculated with MHCC97-H cells, and were treated with stabilizer (as the control of Anlotinib-nanocrystal) or solvent control (as the control of Anlotinib solution).

For the liver orthotopic tumor model, MHCC97-H cells were first cultured and inoculated subcutaneously in nude mice to form tumor tissues. After the tumor tissue is collected, the part with good surface properties of the tumor tissue is stripped off to prepare a tissue micro-block (52–54). Trim the tissue micro-blocks to make the weight basically the same **Supplementary Table 1**). After that, the nude mice were inhaled anesthetized with isoflurane, the aforementioned tumor tissue micro-blocks were inoculated into the nude mice’s liver by open surgery, and finally the nude mice’s wounds were sutured. The drug treatment strategy is basically the same as the above, using microPET to perform live imaging of animals (55) and quantitative analysis of mouse liver images (47, 49): determining the intensity of the microPET image based on the area and pixel density (i.e., brightness) of the image in the liver region in the microPET images; determine the size of the lesion based on the ratio of the area of the lesions to the area of the entire liver organ (47, 49, 54).

### 2.6 Statistical Analysis

The statistical analyses were performed using SAS, version 9.4 (SAS Institute, Inc., Cary, NC, USA) and performed a one-way analysis of variance to compare the means and standard deviations of each treatment method and calculate the p values.

## 3 Research Results

### 3.1 Preparation of Anlotinib Nanocrystals

**Figure 1:**
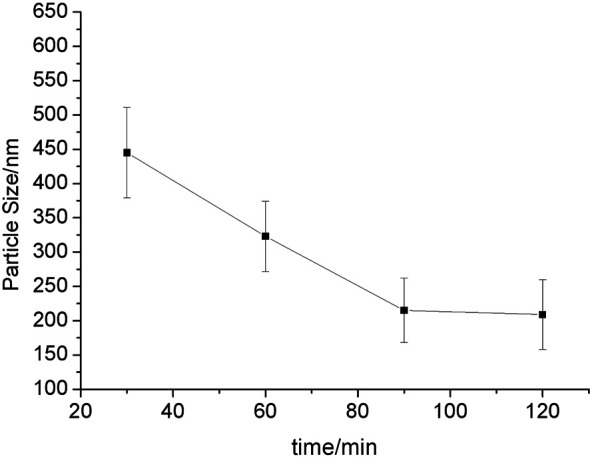
Relationship between particle size and grinding time. The results are shown as line-chart images. The abscissa (X-axis) shows the grinding time points (min) and the ordinate (Y-axis) shows the particle size (nm).

**Figure 2:**
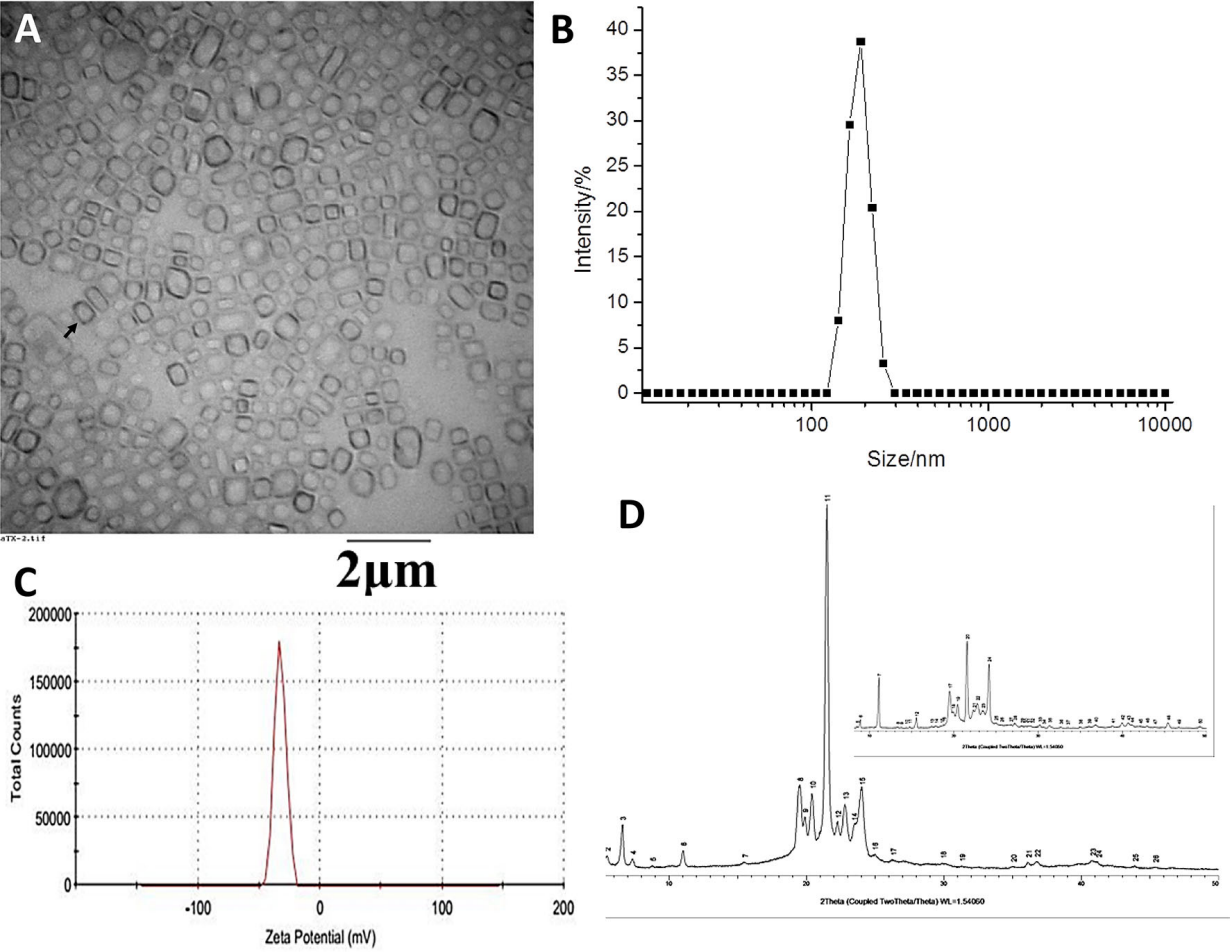
Physical characterization of the anlotinib nanocrystal. **(A)** Transmission Electron Microscope (TEM) images of anlotinib nanocrystal; **(B)** distribution of the anlotinib nanocrystals’ particle sizes; **(C)** zeta potential of the anlotinib nanocrystals; and, **(D)** X-ray diffraction images displaying the anlotinib crystal shape. The black arrow **(A)** indicates the anlotinib nanocrystal particles.

We successfully prepared the anlotinib nanocrystals (**Figures 1**, **2**). As shown in **Figure 1**, the longer the grinding time, the smaller the mean particle size of the anlotinib nanocrystals; and after 90 minutes’ grinding, the smallest nanocrystals reached approximately 200 nm, an appropriate size for intravenous administration. We determined that the optimal formulation process for preparing anlotinib nanocrystals includes the stabilizers Poloxamer 188 and egg yolk lecithin E80 in a mixture ground continuously for 90 to 120 minutes to obtain an anlotinib-nanocrystalline injection preparation with a uniform particle size distribution and an average particle size of approximately 200 nm.

The TEM anlotinib nanocrystal images on **Figure 2A** show the uniform particle size distribution of the anlotinib nanocrystals, with an average particle size of approximately 200 nm (**Figure 2B**). The TEM microscopic morphology analysis results showed well-formed nanocrystals that were regular in shape and uniform in size, without large drug particles (**Figure 2A**). The Zeta potential of the nanocrystals was approximately -30 mV (**Figure 2C**), indicating that the surface of the nanocrystal was negatively charged and that the potential value was moderate, which is beneficial for maintaining the physical stability of the nanocrystal. Moreover, results from an X-ray powder diffraction analysis showed that the crystal structure of anlotinib nanocrystals did not change during the preparation process (**Figure 2D**). The powder diffraction pattern of the bulk drug showed multiple characteristic peaks, and those same characteristic peaks were still present in the nanocrystal preparation, as shown in **Figure 2D**. These results indicate that our efforts to obtain anlotinib nanocrystals were successful.

### 3.2 *In Vitro* Release of Anlotinib Nanocrystals and the Tissue Disposition

**Figure 3:**
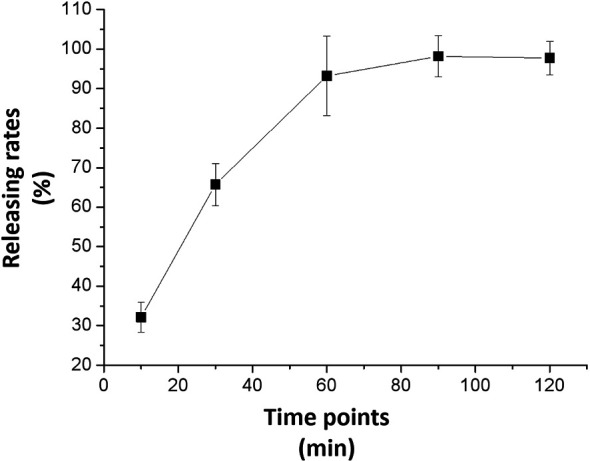
*In vitro* release from the anlotinib nanocrystal. The results are shown as line-chart images. The abscissa (X-axis) shows the releasing time points (min) and the ordinate (Y-axis) shows the releasing rates (%).

**Figure 3** shows the results of the anlotinib nanocrystals’ *in vitro* release. Anlotinib was completely released from the anlotinib nanocrystals within 2 h, and the release rate reached more than 90% within 1 h. These suggest that the prepared anlotinib nanocrystals can effectively release the anlotinib. Moreover, as shown in **Supplementary Figure 1**, Anlotinib-nanocrystals have the characteristic of being selectively enriched in the liver and the selective concentration of Anlotinib after Anlotinib-nanocrystal injected in the liver is beneficial to the treatment of HCC. The accumulation of Anlotinib in the kidney may be due to the excretion and clearance of Anlotinib.

### 3.3 *In Vivo* Tumor Experiments

**Figure 4:**
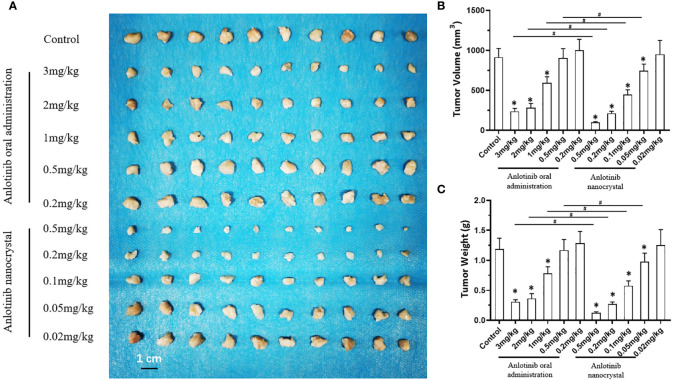
Effect of anlotinib formulation (anlotinib crude suspension or anlotinib nanocrystal preparation) on subcutaneous tumor formation by MHCC97-H cells. MHCC97-H cells were subcutaneously injected into nude mice to generate tumoral tissues. The mice received an oral anlotinib crude suspension or the intravenous anlotinib preparation *via* the tail vein. The results are shown as tumor tissue images **(A)**, tumor volumes **(B)**, or the tumor weights **(C)**. *P < 0.05 *versus* anlotinib with control; ^#^P < 0.05; n = 10 for every group.

We examined the subcutaneous growth of HCC cells to further confirm the antitumor effects of anlotinib nanocrystals on HCC. As shown in **Figure 4**, both the crude oral anlotinib suspension administration (3 mg/kg, 2 mg/kg, 1 mg/kg, 0.5 mg/kg, or 0.2 mg/kg) and the intravenous anlotinib nanocrystal administration (0.5 mg/kg, 0.2 mg/kg, 0.1mg/kg, 0.05mg/kg, or 0.02 mg/kg) inhibited the subcutaneous growth of the HCC cell line, MHCC97-H, in a dose-dependent manner. The intravenous anlotinib nanocrystal injection at a dose of 0.05 mg/kg can inhibit the subcutaneous growth of MHCC97-H cells in nude mice; whereas the dose of orally administered crude anlotinib suspension required to obtain equivalent inhibitory results was 1 mg/kg. Thus, the anlotinib nanocrystal preparation achieved an anti-tumor activity against HCC at very small doses.

**Figure 5:**
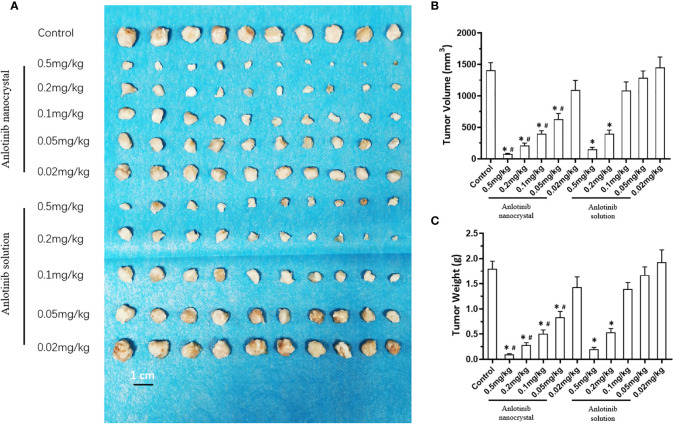
Effect of anlotinib formulation (anlotinib solution or anlotinib nanocrystal preparation) on subcutaneous tumor formation by MHCC97-H cells. MHCC97-H cells were injected subcutaneously into nude mice to generate tumoral tissues. The mice received an intravenous anlotinib solution or the intravenous anlotinib nanocrystal preparation *via* the tail vein. The results are shown as tumor tissue images **(A)**, tumor volumes **(B)**, or tumor weights **(C)**. *P < 0.05 *versus* Anlotinib with control; ^#^P < 0.05 *versus* Anlotinib nanocrystal with Anlotinib solution; n = 10 for every group.

Next, we compared the antitumor effects of the anlotinib nanocrystals injection with the anlotinib solution injection to further confirm the effects of the anlotinib nanocrystal injection preparation. As shown in **Figure 5**, both the intravenous injection of anlotinib nanocrystals or of anlotinib solution inhibited the subcutaneous growth of MHCC97-H cells. However, the antitumor effect of the nanocrystal preparation was better than that if the anlotinib solution.

To further examine the antitumor activation of Anlotinib-nanocrystal, the intrahepatic tumor model was examined. As shown in **Supplementary Figure 2**, the MHCC97-H cells could form the intrahepatic lesions/nodules in nude mice’s liver organs. These lesions could be examined by the microPET imaging (**Supplementary Figure 2**). The anti-tumor activity of the drug was reflected by the intensity of the microPET image and the size of the tumor lesion (**Supplementary Figure 2**). The stabilizer did not have anti-tumor activity (**Supplementary Figure 2**). Tail vein injection of Anlotinib-nanocrystal at a dose of 0.2 mg/kg and Anlotinib solution at a dose of 0.2 mg/kg have the same anti-tumor activity as oral Anlotinib crude suspension at a dose of 3 mg/kg (**Supplementary Figure 2**). These results confirm the advantages of the anlotinib nanocrystal preparation.

### 3.4 Anlotinib Nanocrystal Treatments Caused Less Adverse Effects Than Other Anlotinib Treatments

To examine whether the Anlotinib nanocrystal can also relieve adverse-effects, we examined the animal body weights, main organ weights, and blood routine indicators of the treated animals. As shown in **Table 1**, oral administration of crude anlotinib suspension significantly induced a decrease in hematological markers (leukocyte and red blood cell numbers, hemoglobin content, and platelet counts) and a decrease in body or major organs’ weights (heart weight, liver weight, lung weight, kidney weight or spleen weight) of the nude mice mentioned in **Figure 4**. The intravenous injection of anlotinib nanocrystals produced adverse effects after injection in the mice, but those were milder than the effects observed after oral administration of crude anlotinib suspension (**Table 1**). Moreover, the adverse effects of anlotinib nanocrystals were less severe than those after the intravenous injection of anlotinib solution (**Table 1**). These results further confirm the advantages of the anlotinib nanocrystal preparation.

**Table 1:** The side-effect of Anlotinib-formulations on nude mice’s body weights, organs mass or hematological parameters.

Formulation of Anlotinib	Concentration (mg/kg)	Weights of whole body or organs						hematological parameters			
		Body weight (g)	Heart (mg)	Liver (mg)	Spleen (mg)	Double kidney (mg)	Lung (mg)	Leukocyte (10^9^/L)	Red blood cell (10^12^/L)	Hemoglobin (g/L)	Platelet count (10^9^/L)
untreatment group	–	20.96 ± 2.46	118.01 ± 9.62	659.15 ± 44.83	17.99 ± 0.85	241.17 ± 10.04	162.94 ± 24.676	4.12 ± 0.29	9.53 ± 0.61	155.15 ± 32.76	659.32 ± 12.43
crude Anlotinib suspension	3	10.28 ± 1.01	51.24 ± 3.59	319.80 ± 22.96	8.56 ± 0.14	152.22 ± 27.15	96.59 ± 11.01	1.94 ± 0.25	4.64 ± 0.25	81.13 ± 1.22	347.05 ± 9.73
	2	13.04 ± 0.52	68.96 ± 3.33	401.91 ± 6.99	10.63 ± 0.96	182.79 ± 5.64	108.35 ± 13.99	2.17 ± 0.35	5.42 ± 0.64	90.57 ± 10.44	420.22 ± 6.95
	1	17.85 ± 0.96	83.15 ± 5.30	487.13 ± 26.67	13.28 ± 0.44	197.24 ± 15.60	115.60 ± 7.34	3.02 ± 0.59	6.63 ± 1.76	125.29 ± 4.72	551.67 ± 16.33
	0.5	20.48 ± 0.77	105.95 ± 8.36	599.33 ± 10.43	16.25 ± 1.83	214.64 ± 9.99	131.82 ± 15.78	3.54 ± 0.82	8.09 ± 0.44	137.98 ± 5.80	657.66 ± 14.52
	0.2	20.39 ± 1.41	120.24 ± 13.48	640.62 ± 8.18	18.93 ± 0.15	236.49 ± 22.97	152.95 ± 16.56	4.11 ± 0.61	8.93 ± 0.99	150.55 ± 16.87	641.30 ± 13.14
Anlotinib nanocrystals	0.5	14.41 ± 0.75	73.18 ± 3.48	427.43 ± 10.78	12.56 ± 0.84	171.54 ± 18.67	130.68 ± 13.91	2.41 ± 0.22	5.82 ± 0.47	115.74 ± 9.45	446.01 ± 17.25
	0.2	15.12 ± 1.05	86.88 ± 4.91	495.49 ± 9.22	12.46 ± 1.01	198.55 ± 14.45	145.22.766165	3.14 ± 0.54	5.60 ± 0.63	134.88 ± 5.27	535.24 ± 13.33
	0.1	17.62 ± 0.85	91.34 ± 6.12	535.18 ± 19.97	14.70 ± 0.59	214.61 ± 9.56	150.51 ± 15.64	3.78 ± 0.28	7.55 ± 0.58	147.08 ± 11.68	599.80 ± 18.87
	0.05	19.76 ± 2.47	103.83 ± 7.43	603.96 ± 23.20	16.12 ± 0.71	241.61 ± 34.99	158.24 ± 15.77	4.03 ± 0.76	8.35 ± 0.79	151.20 ± 8.95	633.17 ± 29.25
	0.02	20.32 ± 1.25	119.86 ± 9.05	635.78 ± 17.03	16.33 ± 0.55	233.93 ± 25.37	160.30 ± 4.96	4.14 ± 0.74	9.81 ± 2.01	160.41 ± 25.08	660.02 ± 17.86
Anlotinib solution	0.5	11.02 ± 0.55	48.96 ± 4.98	285.36 ± 34.84	10.13 ± 0.36	130.45 ± 16.88	100.29 ± 10.07	1.00 ± 0.11	3.90 ± 0.78	75.47 ± 12.33	386.59 ± 27.67
	0.2	12.15 ± 2.72	60.71 ± 6.15	385.06 ± 14.73	11.82 ± 0.57	135.96 ± 7.51	108.83 ± 8.12	1.51 ± 0.85	3.99 ± 0.89	87.30 ± 5.94	429.05 ± 29.57
	0.1	14.49 ± 1.70	78.09 ± 4.67	435.80 ± 8.53	12.65 ± 0.52	150.13 ± 3.88	134.50 ± 7.95	2.03 ± 0.62	5.05 ± 0.53	101.74 ± 10.31	520.01 ± 4.44
	0.05	17.58 ± 0.22	90.66 ± 4.42	515.34 ± 8.20	15.98 ± 0.67	162.30 ± 9.44	140.67 ± 9.88	2.95 ± 0.67	7.83 ± 0.78	125.48 ± 15.92	5.88 ± 10.75
	0.02	19.24 ± 1.48	101.50 ± 4.55	560.79 ± 27.86	17.67 ± 1.16	183.08 ± 21.67	159.63 ± 19.71	3.16 ± 0.56	8.65 ± 1.72	143.65 ± 33.10	626.04 ± 31.67

The above results were all using Anlotinib. In order to further confirm the specificity of the system, we also tested the stabilizer and solvent controls. The results are shown in **Supplementary Figure 3**. The stabilizer and solvent controls do not have any anti-tumor activity by themselves. The stabilizer has no obvious toxicity to nude mice, while the solvent control can significantly damage the mice, which is reflected in: the solvent control but not stabilizer induced a decrease in body or major organs’ weights (heart weight, liver weight, lung weight, kidney weight or spleen weight) of the nude mice mentioned in **Supplementary Figure 3** (**Supplementary Table 2**). These results further confirmed the activation of Anlotinib-nanocrystal.

## 4 Discussion

The emergence of molecularly targeted therapy drugs is a milestone in the field of oncology, especially for the treatment of advanced HCC (56, 57). Compared with traditional cytotoxic chemotherapeutics, these agents have higher safety and selectivity profiles, and they have benefited many patients who cannot be cured by surgical operations or who are resistant to common chemotherapeutics (58). Anlotinib can be used clinically for the treatment of locally advanced or metastatic non-small cell lung cancer, but it also offers a new hope for HCC treatment (59, 60). Oral administration has good patient compliance and is usually the preferred route of administration of drugs for the treatment of chronic diseases (61). However, oral administration imposes requirements on the solubility and permeability of the relevant drugs. The ideal oral drugs are those classified as class I by the biopharmaceutics classification system (BCS) (62, 63). For other drugs, different and more effective formulation-techniques need to be adopted to solve solubility/permeability problems (64, 65). Our findings show that after changing its administration route, the anti-tumor effect of the molecularly targeted agent anlotinib was significantly improved (probably due to the elimination of the barrier effect after oral absorption), and this has important implications for this type of agents.

Low solubility and poor oral bioavailability are common problems of some molecularly targeted drugs currently on the market (66). Taking gefitinib or sorafenib as examples, their poor oral absorptions mean large doses (250 mg/person/day for gefitinib and 800 mg/person/day for sorafenib) must be given to achieve therapeutic effects (67–69). Extreme poor water solubility makes intravenous preparations difficult to produce, and advanced intervention and perfusion treatment methods cannot be used. In this study, we used the most advanced nanocrystal technology to prepare anlotinib nanocrystal injection preparations with stable properties and a uniform particle size distribution. Our *in vivo* and *in vitro* evaluation results show that the anlotinib nanocrystal preparation was suitable for intravenous injection and that only 1/10 of the oral dose was needed to achieve similar anti-tumor effects to those of oral gavage in mice. Thus, by changing the route of administration of anlotinib to the intravenous route, we probably eliminated the absorption barrier at the gastrointestinal tract and thereby greatly improved the drug’s anti-tumor effects.

## 5 Conclusion

The anlotinib nanocrystal injection prepared during our experiments targeted the HCC tumor well, and its anti-tumor effects were better than those of the oral crude suspension. Our results show that the preparation of a new drug delivery system based on new nano-preparation technology can change the traditional route of drug delivery to maximize its strengths and prevent weaknesses. In the case of anlotinib, the new preparation improved its anti-tumor effects, an important reference with implications for other small molecule kinase inhibitors.

## Data Availability Statement

The original contributions presented in the study are included in the article/**Supplementary Material**. Further inquiries can be directed to the corresponding authors.

## Ethics Statement

The animal study was reviewed and approved by Animal Ethics Committee of the Fifth Medical Center, General Hospital of Chinese PLA.

## Author Contributions

ML, ZH, and BY: concept, design, statistics, data collection, manuscript writing, and final approval. ZH and BY: design, statistics, and data collection. HS, YC, QJ, and CL: concept and data collection. HS, YC, QJ, and CL: statistics and manuscript writing. HS, YC, QJ, and CL: statistics and data collection. HS, YC, QJ, and CL: statistics and data collection. ML, ZH, and BY: concept, design, statistics, data collection, manuscript writing, final approval. All authors contributed to the article and approved the submitted version.

## Conflict of Interest

The authors declare that the research was conducted in the absence of any commercial or financial relationships that could be construed as a potential conflict of interest.

## Publisher’s Note

All claims expressed in this article are solely those of the authors and do not necessarily represent those of their affiliated organizations, or those of the publisher, the editors and the reviewers. Any product that may be evaluated in this article, or claim that may be made by its manufacturer, is not guaranteed or endorsed by the publisher.

## Supplementary Material

Supplementary Figure 1 Tissue distribution of Anloitnib in rats after Anlotinb-nanocrystals injection. After injection of Anlotinib-nanocrystal into the tail vein of the rat, the various organs of the animal were collected at different time points for testing to determine the content of Anlotinib. **(A)** Histogram; **(B)** line chart.

Supplementary Figure 2 Effect of anlotinib formulation (anlotinib crude suspension, anlotinib anlotinib solution or anlotinib nanocrystal preparation) on subcutaneous tumor formation by MHCC97-H cells. MHCC97-H cells were injected subcutaneously into nude mice to generate tumoral tissues and then prepared as the intrahepatic tumor model. The mice received an oral administration of anlotinib crude suspension (3mg/kg dose) an intravenous anlotinib solution (0.2mg/kg dose) or the intravenous anlotinib nanocrystal preparation (0.2mg/kg) *via* the tail vein. The results are shown as liver organs images with intrahepatic lesions **(A)**, images of microPET **(A)**, or the quantitative results **(B, C)**. *P < 0.05; n = 6 for every group The white arrows in **(A)** respectively refer to the liver region in the microPET image or the Lesions in the liver organs; Anli-oral: anlotinib crude suspension (3mg/kg dose); Anl-Sol: anlotinib solution (0.2mg/kg dose); Anl-nano: the intravenous anlotinib nanocrystal preparation (0.2mg/kg).

Supplementary Figure 3 The effect of stabilizer or Solvent control on the subcutaneous growth of MHCC97-H cells in nude mice. MHCC97-H cells were injected subcutaneously into nude mice to generate tumoral tissues. The mice received an intravenous Solvent control of anlotinib solution or the intravenous stabilizer of anlotinib nanocrystal preparation *via* the tail vein mentioned in **Figure 5**. The results are shown as tumor tissue images **(A)**, tumor volumes **(B)**, or tumor weights **(C)**. n = 6 for every group.
