# Supplementary figures and images for: Novel Nanocrystal Injection of Insoluble Drug Anlotinib and Its Antitumor Effects on Hepatocellular Carcinoma
Source: Front Oncol. 2021 Dec 2;11:777356. doi: 10.3389/fonc.2021.777356 (PMC8674816; doi:10.3389/fonc.2021.777356)

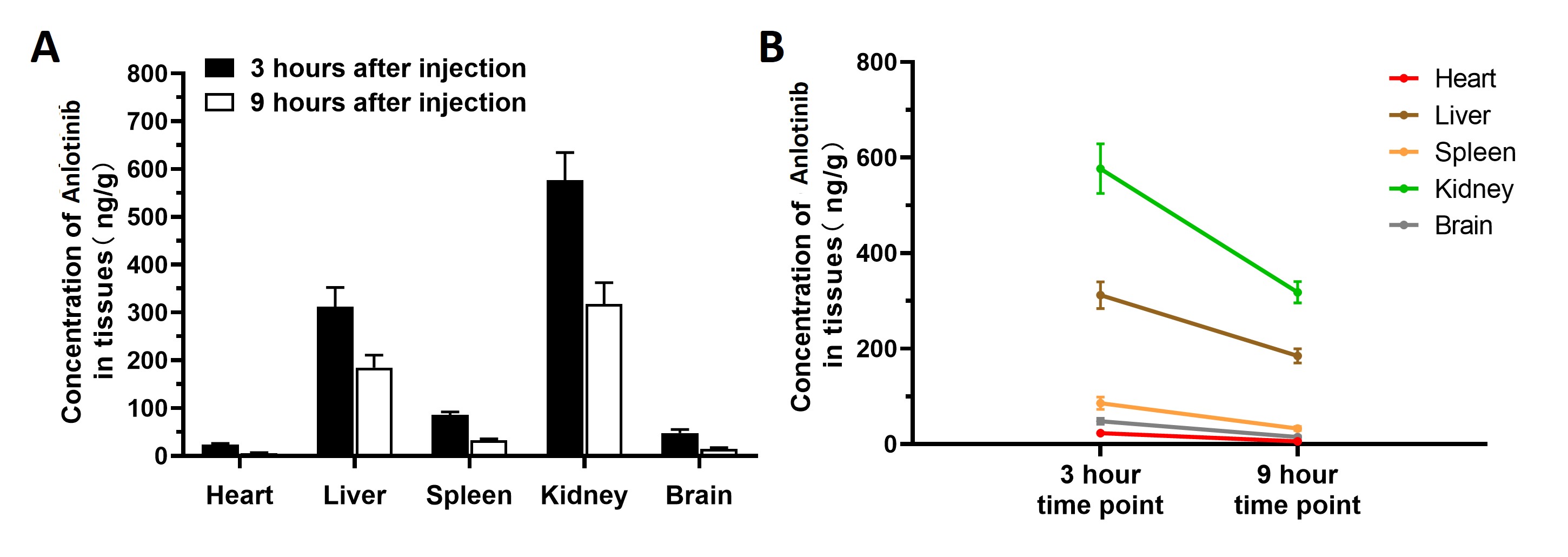

Supplement: Supplementary Figure 1 — Tissue distribution of Anloitnib in rats after Anlotinb-nanocrystals injection. After injection of Anlotinib-nanocrystal into the tail vein of the rat, the various organs of the animal were collected at different time points for testing to determine the content of Anlotinib. (A) Histogram; (B) line chart. [file Image_1.jpeg]

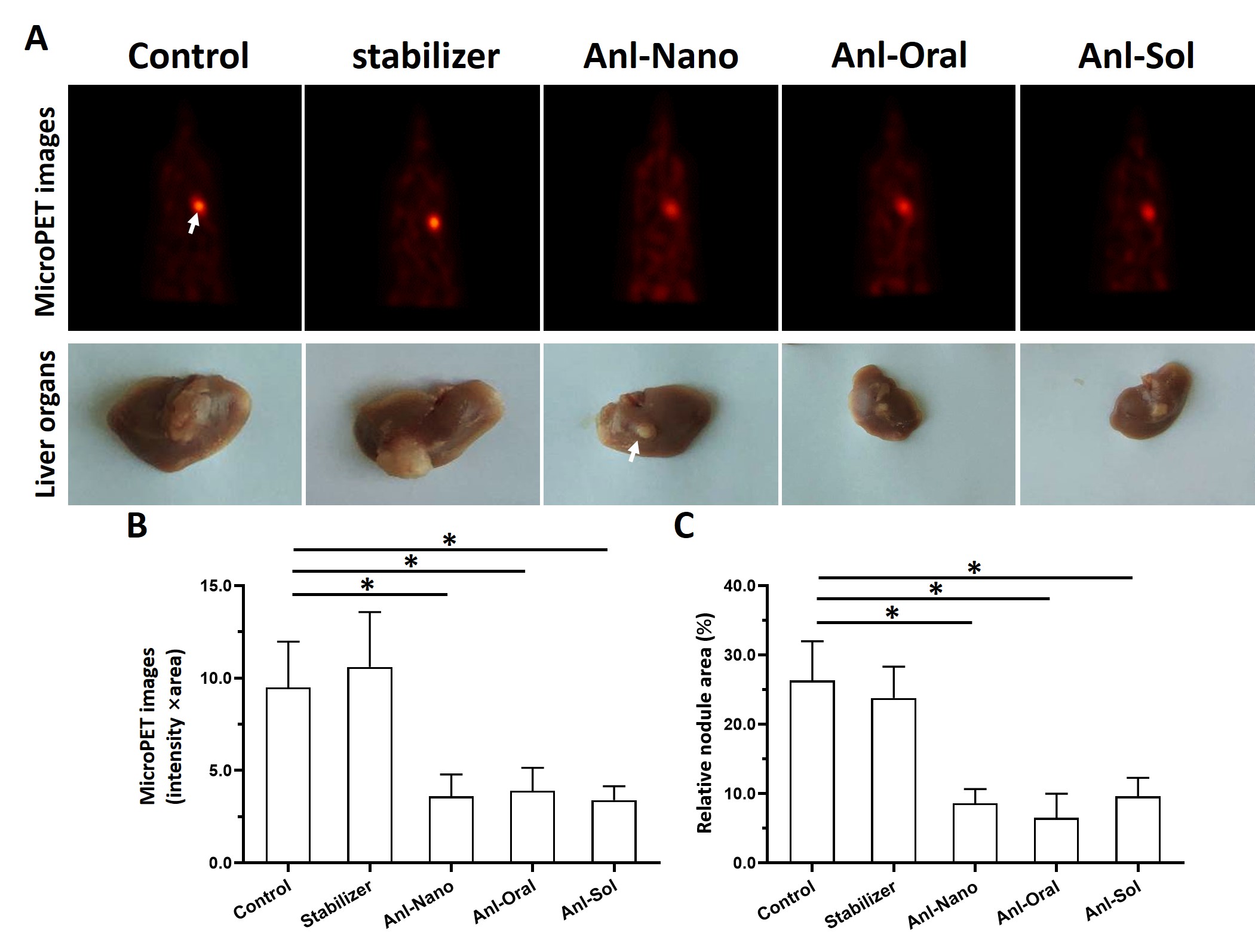

Supplement: Supplementary Figure 2 — Effect of anlotinib formulation (anlotinib crude suspension, anlotinib anlotinib solution or anlotinib nanocrystal preparation) on subcutaneous tumor formation by MHCC97-H cells. MHCC97-H cells were injected subcutaneously into nude mice to generate tumoral tissues and then prepared as the intrahepatic tumor model. The mice received an oral administration of anlotinib crude suspension (3mg/kg dose) an intravenous anlotinib solution (0.2mg/kg dose) or the intravenous anlotinib nanocrystal preparation (0.2mg/kg) via the tail vein. The results are shown as liver organs images with intrahepatic lesions (A), images of microPET (A), or the quantitative results (B, C). *P < 0.05; n = 6 for every group The white arrows in (A) respectively refer to the liver region in the microPET image or the Lesions in the liver organs; Anli-oral: anlotinib crude suspension (3mg/kg dose); Anl-Sol: anlotinib solution (0.2mg/kg dose); Anl-nano: the intravenous anlotinib nanocrystal preparation (0.2mg/kg). [file Image_2.jpeg]

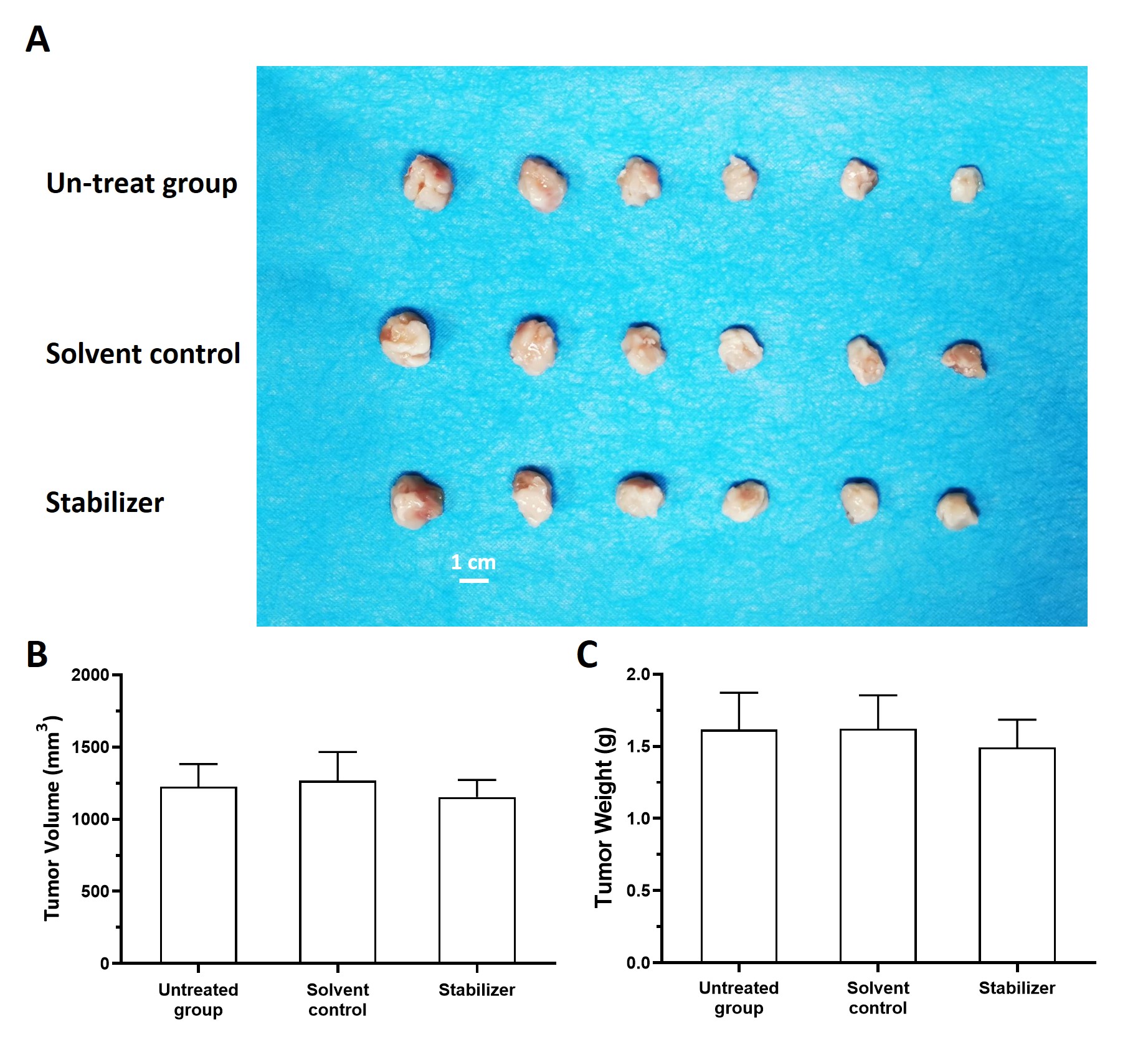

Supplement: Supplementary Figure 3 — The effect of stabilizer or Solvent control on the subcutaneous growth of MHCC97-H cells in nude mice. MHCC97-H cells were injected subcutaneously into nude mice to generate tumoral tissues. The mice received an intravenous Solvent control of anlotinib solution or the intravenous stabilizer of anlotinib nanocrystal preparation via the tail vein mentioned in Figure 5 . The results are shown as tumor tissue images (A), tumor volumes (B), or tumor weights (C). n = 6 for every group. [file Image_3.jpeg]
