# Supplementary material for: Novel Nanocrystal Injection of Insoluble Drug Anlotinib and Its Antitumor Effects on Hepatocellular Carcinoma
Source: Front Oncol. 2021 Dec 2;11:777356. doi: 10.3389/fonc.2021.777356 (PMC8674816; doi:10.3389/fonc.2021.777356)
Supplement: Supplementary file 4 [file Table_1.doc]

Supplemental Table 1 The weights of micro-blocks of tumor tissues formed by MHCC97-H to prepare the intrahepatic tumor model

| mice No. | control | stabilizer | Anl-nano | Anl-Oral | Anl-Sol |
| --- | --- | --- | --- | --- | --- |
| 1 | 3.15 | 3.52 | 3.17 | 3.24 | 3.50 |
| 2 | 3.22 | 3.24 | 3.51 | 3.40 | 3.43 |
| 3 | 3.42 | 3.07 | 3.00 | 3.42 | 2.92 |
| 4 | 3.31 | 3.09 | 3.18 | 3.17 | 3.30 |
| 5 | 3.17 | 3.44 | 3.41 | 3.39 | 3.47 |
| 6 | 3.46 | 3.43 | 3.30 | 2.95 | 2.93 |
