# Supplementary material for: Novel Nanocrystal Injection of Insoluble Drug Anlotinib and Its Antitumor Effects on Hepatocellular Carcinoma
Source: Front Oncol. 2021 Dec 2;11:777356. doi: 10.3389/fonc.2021.777356 (PMC8674816; doi:10.3389/fonc.2021.777356)
Supplement: Supplementary file 5 [file Table_2.doc]

Supplemental Table 2 The effect of stabilizer or Solvent control on nude mice’s body weights or organs mass

| Weights | control | stabilizer | Solvent control |
| --- | --- | --- | --- |
| Body weight (g) | 21.38±0.78 | 21.27±1.73 | 19.75±0.90 |
| Heart (mg) | 119.04±18.57 | 120.05±28.11 | 99.67±11.62 |
| Liver (mg) | 640.57±25.35 | 634.20±34.81 | 576.04±23.24 |
| Spleen (mg) | 18.08±0.44 | 17.84±1.44 | 16.49±0.50 |
| Double kidney (mg) | 236.94±9.63 | 226.78±19.37 | 195.74±31.55 |
| Lung (mg) | 158.33±15.67 | 160.94±21.49 | 140.41±12.41 |
